# Accurate genome relative abundance estimation for closely related species in a metagenomic sample

**DOI:** 10.1186/1471-2105-15-242

**Published:** 2014-07-16

**Authors:** Michael B Sohn, Lingling An, Naruekamol Pookhao, Qike Li

**Affiliations:** Interdisciplinary Program in Statistics, University of Arizona, Tucson, AZ 85721 USA; Department of Agricultural and Biosystems Engineering, University of Arizona, Tucson, AZ 85721 USA

**Keywords:** Metagenomics, Alignment similarity, Genomic similarity, Closely related species

## Abstract

**Background:**

Metagenomics has a great potential to discover previously unattainable information about microbial communities. An important prerequisite for such discoveries is to accurately estimate the composition of microbial communities. Most of prevalent homology-based approaches utilize solely the results of an alignment tool such as BLAST, limiting their estimation accuracy to high ranks of the taxonomy tree.

**Results:**

We developed a new homology-based approach called Taxonomic Analysis by Elimination and Correction (TAEC), which utilizes the similarity in the genomic sequence in addition to the result of an alignment tool. The proposed method is comprehensively tested on various simulated benchmark datasets of diverse complexity of microbial structure. Compared with other available methods designed for estimating taxonomic composition at a relatively low taxonomic rank, TAEC demonstrates greater accuracy in quantification of genomes in a given microbial sample. We also applied TAEC on two real metagenomic datasets, oral cavity dataset and Crohn’s disease dataset. Our results, while agreeing with previous findings at higher ranks of the taxonomy tree, provide accurate estimation of taxonomic compositions at the species/strain level, narrowing down which species/strains need more attention in the study of oral cavity and the Crohn’s disease.

**Conclusions:**

By taking account of the similarity in the genomic sequence TAEC outperforms other available tools in estimating taxonomic composition at a very low rank, especially when closely related species/strains exist in a metagenomic sample.

**Electronic supplementary material:**

The online version of this article (doi:10.1186/1471-2105-15-242) contains supplementary material, which is available to authorized users.

## Background

Metagenomics is the study of microbes by analyzing the entire genomic contents extracted directly from an environmental sample. Its growth has been greatly encouraged by the rapid advances in Next Generation Sequencing (NGS) technologies, which deliver massive volumes of sequence data at relatively low cost and fast turnaround time [1–3]. An essential prerequisite for metagenomic analysis is to unriddle the taxonomic composition of the microbial community in a given sample. It is generally accomplished by aligning sequencing reads against databases of known genomes or of phylogenetic marker genes [4], which is known as the homology-based approach. A challenge is that many microbes in an environmental sample share the similarity in the genomic sequence, and this intrinsic complexity of metagenomic samples makes it extremely difficult, if not impossible, to accurately estimate the taxonomic composition, especially at low ranks of taxonomy tree, such as the species/strain level.

One early approach to estimate taxonomic composition of metagenomic samples is to use the Lowest Common Ancestor (LCA) method implemented in MEGAN [5], in which a sequencing read matching with multiple genomes is assigned to their lowest common ancestor, lowering the false positive rate at the cost of the specificity of assignment. In order to improve the specificity, various approaches have been developed [6–11].

One recent homology-based approach, GASiC [10] utilizes the similarity in the genomic sequence to estimate taxonomic composition at the species level. To this end, it estimates the similarity between genomes by simulating a set of reads from each genome in a given sample and aligning it to the every genome in the sample individually. With the estimated similarity in the genomic sequence, it corrects the species abundance parsed from the result of an alignment tool such as Bowtie2 [12] using a non-negative LASSO approach [13]. However, this approach requires prior information about genomes in a given sample in order to construct a matrix of the similarity among the genomes so that it can estimate the relative abundance of the genomes. Therefore, it is not very suitable for metagenomic samples whose contents are usually unknown, yet need to be identified.

**Figure 1:**
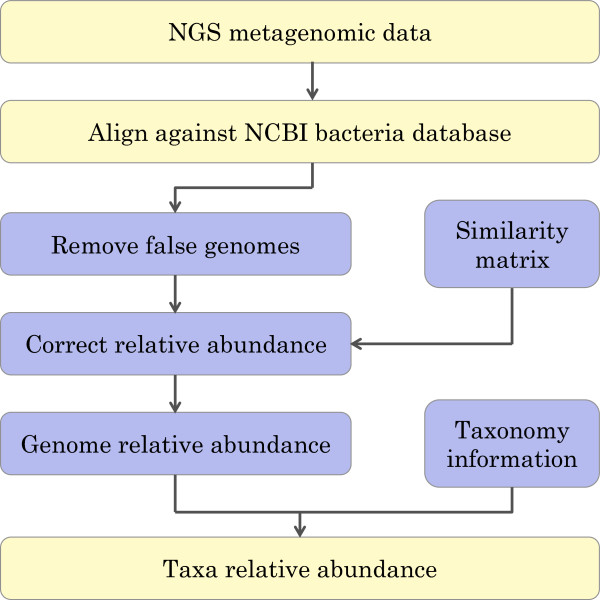
**Flow chart of TAEC’s workflow.** The light yellow colored blocks are implemented by a user and the light blue colored blocks are internally implemented by TAEC. Note: the bacteria database could be replaced with virus or other types of databases if needed.

Another recent homology-based approach, TAMER [11] proposed a mixture model to estimate the proportion of sequence reads assigned to each genome while accommodating information of sequencing alignments. The parameters of the mixture model estimated by the EM algorithm [14] are used to assign each read to the most probable genome. The output of TAMER is at the species/strain level. However, estimated abundance is not accurate for highly similar genomes (genomes sharing high similarity in their genomic sequences) because of their high correlation, which cannot be captured by the mixture model.In this paper, we propose a new homology-based approach, Taxonomical Analysis by Elimination and Correction (TAEC). This approach consists of two main stages: the elimination stage and the correction stage. In the elimination stage, we remove genomes whose presence is most likely due to the presence of similar genomes in a sample. In the correction stage, we correct the abundance of each genome remaining after the elimination stage by utilizing the similarity among the genomes in a system of linear equations. The overall workflow of TAEC is shown in Figure 1.

TAEC is similar to GASiC in that both methods use the similarity in the genomic sequence among genomes to correct the abundance estimation. However, TAEC is quite different from GASiC in that it utilizes the uniqueness of genome to remove possible false genomes before the correction of abundance. TAMER is fundamentally different from the two methods: it does not consider the similarity between genomes in the assignment of reads. It only depends on the estimated post probability. In other words, when a read is mapped to multiple genomes in a BLAST output, it will be generally assigned to the most abundant genome (obtained in the parameter estimation step) regardless of similarity between genomes.

We tested TAEC on various simulated datasets and compared its performance with that of the aforementioned two methods, which were already demonstrated to outperform many other methods [10, 11]. TAEC showed consistent performance regardless of complexity of metagenomic samples, even on a sample containing highly similar genomes, where the other methods showed poor performance. We also applied TAEC to two real metagenomic samples collected from human mouth [15] and human gut [16] and obtained their taxonomic compositions at the species/strain level, providing an interesting insight into the samples.

## Methods

### Input data and reference database

As other homology-based methods, DNA sequences or reads in a sample are compared against databases of known genomes using a sequence alignment tool such as BLAST in the preliminary stage. TAEC is then used to estimate the taxonomic composition of the sample by utilizing the similarity in the genomic sequence. In this research, we performed sequence alignments against the NCBI bacteria database using BLASTN to estimate the similarity among genomes. Thus, the input data should be an alignment result from BLASTN against the NCBI bacteria database [17] for the current version of TAEC. However, our approach is not restricted to BLASTN nor the NCBI bacteria database. It can be used with any alignment tools and reference databases.

### Similarity estimation

For a reference database that contains *N*_0_ genomes, the method described below is used to estimate the similarity in the genomic sequence between any two genomes in the database. A set of *K*_0_ random reads for each genome *g*_*j*_ is generated and aligned against the reference database, where *j* = 1,⋯,*N*_0_. The reads are then assigned to genomes based on the alignment score. In cases where a read *r*_*i*_ is aligned to multiple genomes, *r*_*i*_ is assigned to the genomes whose alignment scores are greater than or equal to *α* · max*j* (*s*_*ij*_), where *α* ∈ [0,1] and *s*_*ij*_ is the alignment score of *g*_*j*_ for *r*_*i*_, *i* = 1,⋯,*K*_0_. How to determine the value of *α* depends on the length of reads and the complexity of sample data: shorter reads and more complex datasets require higher value of *α* to distinguish highly similar genomes. The ratio  between the numbers of reads assigned to *g*_*j*_ and  can present the probability that reads originating from *g*_*j*_ can be assigned to , or , where *n*_*j*_ denotes the number of reads assigned to *g*_*j*_. This ratio is used as the similarity in the genomic sequence between genomes to build a similarity matrix *W* for all genomes in a reference database.

### Elimination stage

Many genomes share more or less similarity in the genomic sequence but each genome has its unique regions, which differentiate it from other genomes. Therefore, if a genome is truly present in a sample, there must be some reads uniquely assigned to it as long as the depth of coverage is high enough. We utilize this fact of uniqueness to identify genomes whose presence in the result of an alignment tool is most likely due to the similarity in the genomic sequence to the true genomes in a sample.

To this end, each read is assigned to genome(s) with the highest alignment score, and a binary *K*×*N* matrix *A* is created with its entry *a*_*ij*_= 1 if *r*_*i*_ is assigned to *g*_*j*_ and *a*_*ij*_= 0 otherwise, where *K* is the number of reads and *N* is the number of genomes present in the result of an alignment tool. For example, the below is the BLAST output for a small set of six reads:


Let {*a*_*j*_} be the columns of A, and *a*_(*j*)_ be the column with max*j* ≤ *l* ≤ *N*||*a*_*l*_||_1_, where ||*a*_*l*_||_1_ is *L*^1^-norm of *a*_*l*_, which corresponds to the total number of reads assigned to the genome *g*_*l*_. To identify the genomes to which no reads are uniquely assigned, with *A*_0_ = *A* we inductively solve the following equation (a simple example of how Eq. (1) works and an equivalent iterative algorithm are provided in Additional file 1):

1

until we get the column *j*_0_ which satisfies , where (*X*)_+_ is a matrix with entries equal to max(*x*_*ij*_,0), *P*_*j*_ a permutation matrix that permutes the column *a*_(*j*)_ with the column *a*_*j*_, and *S*_*j*_ a matrix that subtracts the column *a*_(*j*)_ from each of the columns *a*_*l*_ for *l* > *j*. Now, the genomes represented by the columns *a*_*j*_ for *j* ≥ *j*_0_ can be removed since no reads is uniquely assigned to them, which implies that their presence is mainly due to the similarity in the genomic sequence to the true genomes in a sample. Thus, for the example above *A*_*j*_ becomes as below, i.e., only G3 and G4 are possible true genomes.


In practice, reads can be assigned to some random genomes due to sequencing and alignment errors so the stopping criterion for Eq. (1) can be relaxed such that , where *c* is a user defined minimum number of reads for a genome to be included in the subsequent analysis. The whole elimination procedure can be iterated using non-parametric bootstrap [18]. In the bootstrap, the number of occurrences of  is used as a criterion to decide whether the genome *g*_*j*_ is a false genome: if it exceeds a user defined number, *g*_*j*_ is considered as a false genome and removed.

### Correction stage

In the elimination stage, the uniqueness of genomes is utilized to remove false genomes, disregarding accuracy in the number of reads assigned to each genome. In the example data genomes of G1 and G2 are removed. In the correction stage, the number of reads assigned to each genome remaining after the elimination stage (i.e., G3 and G4) is corrected using the similarity matrix *W* in a system of linear equations.

In this stage, to be consistent with the estimation of the similarity, we use *α*× max*j*(*s*_*ij*_) as a minimum alignment score to reassign a read to the remaining genomes, where *s*_*ij*_ is the alignment score of a genome *g*_*j*_ for a read *r*_*i*_. That is, *r*_*i*_ is assigned to the genomes whose alignment scores are greater than or equal to *α*× max*j*(*s*_*ij*_). Let *b*_*j*_ denote the number of reads assigned to the genome *g*_*j*_ in this way, and *t*_*j*_ be the number of reads assigned to *g*_*j*_ only due to its own presence, which we want to find. Suppose the number of remaining genomes after the elimination stage is *m*. Then, the number of reads assigned to each genome can be given by the following equations:
2

where  is the similarity between *g*_*j*_ and , that is, the (*j*,*j*^′^) entry of the similarity matrix *W*. Since no genome has the perfect similarity to other genomes, or  for all *j*≠*j*^′^, the inverse of *W*^*T*^ exists. Thus, the corrected abundance for each genome can be obtained by solving
3

where *W*^*T*^ is the transpose of *W*, *t* = (*t*_1_,*t*_2_,...,*t*_*m*_)^*T*^ and *b* = (*b*_1_,*b*_2_,...,*b*_*m*_)^*T*^. If *t*_*j*_< 0 for some *j*, Eq. (3) is repeated after removing the genomes *g*_*j*_ until *t*_*j*_> 0 for all *j* since the number of reads cannot be negative.

### Implementation of methods used in TAEC

For the genomes excluding plasmids in the NCBI Bacteria database, we created 4 similarity matrices, one for each of the most common read lengths: 100 bp, 250 bp, 500 bp and 1000 bp. We used 30,000 reads for each genome to estimate the similarity in the genomic sequence among genomes, that is *K*_0_= 30,000, and set 0.001 as a threshold for the similarity between genomes (i.e., if the similarity between two genomes is less than 0.1%, it is set to 0). The detailed information about the selection of *K*_0_ and a threshold for the similarity is provided in Additional file 1.

**Figure 2:**
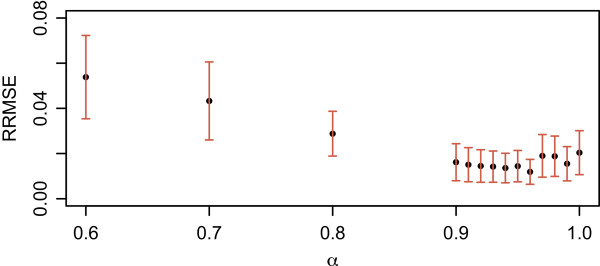
**RRMSE vs.** ***α*** **for read length of 250 bp.** The mean of RRMSE for 40 samples at different *α*. The error bars represents 95% confidence interval. The smallest mean and variance of RRMSE occurs at *α* = 0.96.

In the selection of an optimal value for *α*, we simulated 40 different samples, in each of which we used 100,000 reads of 250 bp originating from 5, 10 or 20 randomly selected genomes at various relative abundance ratios, which were randomly selected such that the ratio between the least and the most abundant genomes can be up to 1:20. We then computed the relative root mean squared error (RRMSE) Eq. (4), defined in the result section, for each sample at different values of *α*. As shown in Figure 2, any value *α*≥0.90 could be chosen since there is no statistically significant difference in the mean of RRMSE at the 95% confidence level. We selected *α*=0.96 since the smallest mean and variance of RRMSE occurs at this value. The value of *α* also depends on the length of reads but not as much as on the complexity of a sample so the gain of accuracy by choosing a different value of *α* for a different read length is marginal (see Additional file 2: Table S1). Thus, we used the same value of *α* for all the similarity matrices.

Since we set 0.001 as the similarity threshold, we could not determine whether the presence of a genome in the elimination stage is due to its true presence or its undetectable similarity to the most abundant genome if its relative abundance is less than 0.1% of the most abundant genome. However, the most abundant genome in the elimination stage is overestimated unless its similarity to other genomes in a sample is zero. Thus, we used 0.05% instead of 0.1% as a cut-off in the decision of which genomes are falsely present in order to minimize false elimination of true low abundant genomes. In the bootstrap, any genome whose abundance is less than 0.05% of the most abundant genome in more than 5% of the bootstrap samples was eliminated. The method developed for the elimination stage is implemented in C as an extension to R to minimize the computation time, and the method for the correction stage is implemented in R. The single run time for an input data of 1.1 GB takes about 1 minute on a laptop with dual core 1.8 Ghz CPU and 4 GB of memory, and each additional run time for the bootstrap takes about 35% of the single run time. The TAEC package is available for download at http://cals.arizona.edu/~anling/software.htm.

## Results

We first tested TAEC on three simulated datasets to evaluate estimation accuracy and to compare with the other methods. We then applied it on two real metagenomic samples to analyze the taxonomic composition of each sample. In the comparison, we used three commonly used error measures [9]: relative root mean squared error (RRMSE), average relative error (AVGRE) and maximum relative error (MAXRE), which are given by
456

where *N* is the number of the true genomes in a sample, *t*_*i*_ the estimated number of reads assigned to genome *i* and *τ*_*i*_ the true number of reads originating from genome *i*. For each of the following studies, we used 100 bootstrap samples in the elimination stage.

### Simulation study I - FAMeS datasets

**Figure 3:**
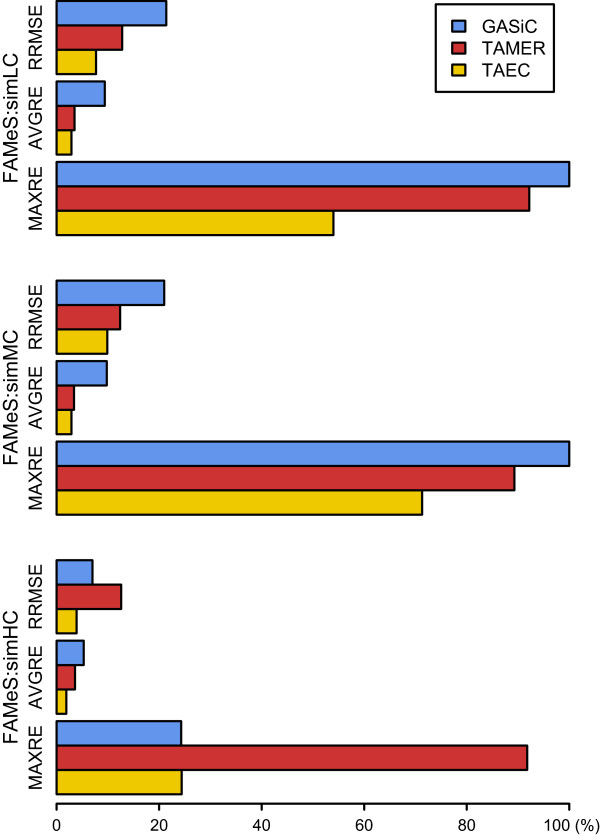
**Estimation accuracy comparison on FAMeS datasets.** The performance of three methods on the FAMeS datasets is compared by the three error measures: RRMSE, AVGRE and MAXRE.

The FAMeS datasets [19] consist of three artificial metagenomic datasets containing shotgun sequencing reads from 113 genomes. These datasets are named ‘simLC’, ‘simMC’ and ‘simHC’ based on abundance complexity: the simLC dataset contains one dominant genome with many low abundant genomes, the simMC dataset contains a few dominant genomes with many low abundant genomes and the simHC dataset contains no dominant genomes. These datasets were used in the GASiC paper [10]. Among 113 genomes in the FAMeS datasets, we used 106 genomes that are contained in the NCBI Bacteria database and compared the performance of TAEC on these datasets with GASiC and TAMER.In the comparison with GASiC, we ignored the p-value that GASiC uses to determine whether a genome is truly present in a sample because only 4 genomes have the p-value less than 0.05 for the simLC and the simMC datasets and 22 for the simHC dataset. The comparisons of estimation accuracy are shown in Figure 3. TAEC yields the lowest errors for all the datasets. In particular, it performs very well on the simHC dataset in which the depth of coverage for each genome is sufficiently high. GASiC, which also uses the similarity between genomes, shows significant improvement on the simHC dataset as well. However, TAMER does not benefit from the increase in the depth of coverage.

### Simulation study II - MetaSim datasets

The MetaSim datasets [20] also consist of three metagenomic datasets named ‘simLC’, ‘simMC’ and ‘simHC’. However, reads in each dataset are simulated by a sequencing simulator, MetaSim, and the name of each dataset is based on the number of genomes in the dataset: the simLC dataset contains 2 genomes, the simMC dataset contains 9 genomes and the simHC dataset contains 11 genomes. These datasets, each of which contains 150,000 reads of length 100 bp, were reproduced using the same parameters used in the MetaSim and the TAMER papers [11, 20] to compare estimation accuracy.

**Figure 4:**
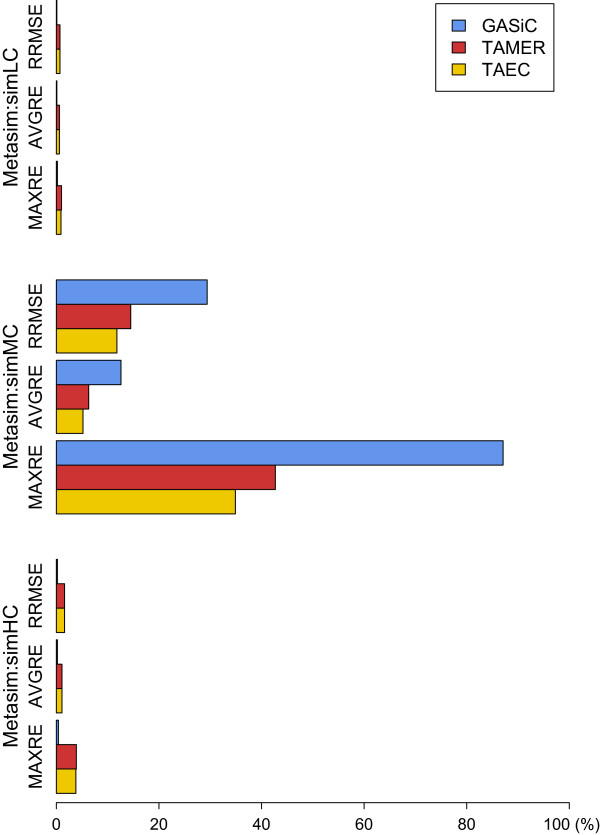
**Estimation accuracy comparison on MetaSim datasets.** The performance of three methods on the Metasim datasets is compared by the three error measures: RRMSE, AVGRE and MAXRE.

All approaches, as shown in Figure 4, perform well on the simLC and the simHC datasets in which all the genomes are very different from each other or the similarity between all genomes is very small. Even MAXREs for all approaches are less than 5% on these datasets. However, for the simMC dataset that contains two relatively similar genomes, *Escherichia coli str. K-12 substr. MG1655* and *Shigella dysenteriae Sd197*, the performance of all approaches deteriorate, but TAEC by the least degree.

**Figure 5:**
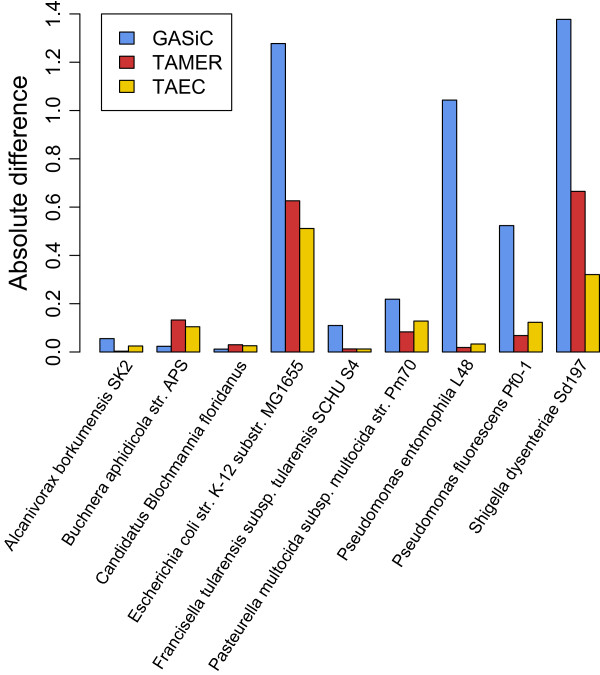
**Details of accuracy on simMC dataset.** The performance of three methods on the Metasim MC dataset is compared by the absolute difference of relative abundance in percentage between the true and the estimated composition.

As shown in Figure 5, which presents the absolute difference between the true and the estimated relative abundance of each genome in percentage, the common sources of high errors for all three methods are *Shigella dysenteriae* and *E. coli*. It is due to the very different relative abundance for the similar genomes (*Shigella dysenteriae* is only about 5% of *E. coli*). For both GASiC and TAEC a small fluctuation in the similarity can cause significant impact on the number of reads for the less abundant genome. The performance of TAEC is less sensitive to difference in relative abundance for similar genomes because of the optimum value of *α*: the similarity between *Shigella dysenteriae* and *E. coli* estimated by TAEC is much lower than that estimated by GASiC, reducing the effect of fluctuation in the similarity. This may be the same reason that GASiC shows large errors on the estimation of *Pseudomonas entomophila* and *Pseudomonas fluorescens*.

### Simulation study III

The last simulation study is motivated by the findings in the preceding simulation study where genomes in a sample are highly similar. The first sub-study was conducted to show the necessity of the similarity information to estimate the taxonomic composition accurately and to show the capacity of TAEC to perform at the species/strain level. Two artificially simulated datasets using MetaSim contain three strains of *Escherichia coli*: *Escherichia coli str. K-12 substr. MG1655*, *Escherichia coli 0103:H2 str. 12009* and *Escherichia coli B str. REL606*. Each of the two datasets contains 150,000 reads of 100 bp from the three strains, but one at the same relative abundance ratio (1:1:1) and the other at different relative abundance ratios (the ratio of 1:2:3).

**Figure 6:**
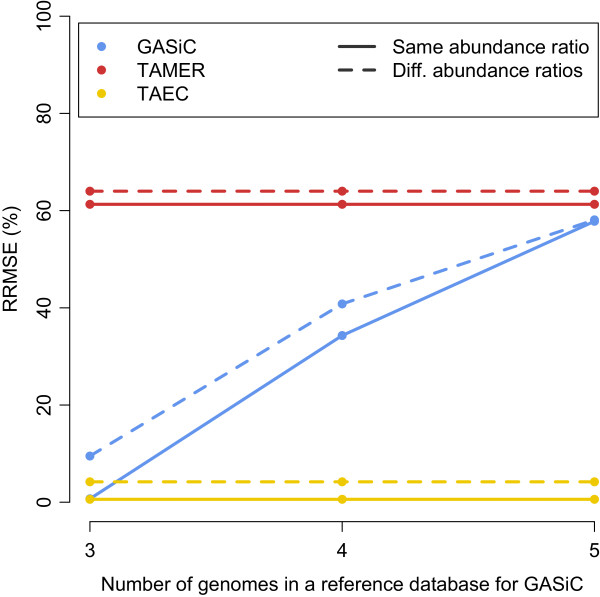
**Estimation accuracy comparison on** ***E. Coli*** **dataset of 3 strains.** The performance of the three methods on the two samples that contain three *E. coli* strains, one at the same relative abundance ratio and the other at different relative abundance ratios, is compared by RRMSE as the number of false genomes in a reference database for GASiC increases. For TAMER and TAEC the reference database is kept same, i.e., NCBI bacteria database.

As GASiC needs to create a reference database we consider three types of database for GASiC: 1) only 3 true genomes 2) additional false genome *Escherichia coli DH1* and 3) adding another false genome *Escherichia coli DH10B*. Even though TAMER and TAEC use the NCBI bacteria database, we just display the performance results of three methods in the same plot, Figure 6. The RRMSEs for TAMER are very high, showing its limitations on the sample that contains very similar genomes; the RRMSE by GASiC dramatically increases when more false (similar) genomes are included in the database but it performs well when only the true genomes are included in the reference database. TAEC outperforms these two methods in this study, showing its consistent performance regardless of the complexity of a sample. These results are at the strain level and can be summarized to a higher level, e.g., species level. At the species level (Additional file 3: Figure S1) the performances of TAEC and TAMER are comparable, and both of them outperform GASiC when false genomes are contained in the reference database.

**Figure 7:**
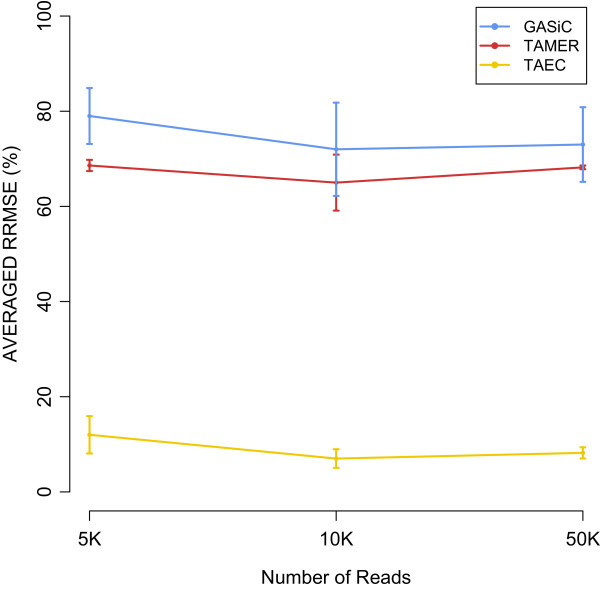
**RRMSE vs. Number of reads: Three** ***E. coli*** **strains at the relative abundance in the ratio of 1:5:10.**

The second sub-study is about the effect of depth of coverage on the accuracy of estimation. We simulated samples containing the same three *E. coli* strains at different sample sizes (i.e., the number of reads) to analyze the effect of depth of coverage on the accuracy of estimation. For TAMER and TAEC, the entire NCBI bacteria database was used for alignment while for GASiC just five *E. coli* strains, the three true and two false ones (mentioned above), were used in the reference database. As shown in Figure 7 and Additional file 4: Figure S2 and Additional file 5: Figure S3, TAMER and GASiC show very large RRMSE and do not benefit from the increase in the sample size. On the contrary, TAEC shows small RRMSE and benefits from the increase in the sample size.

### Oral metagenomic datasets

The microbial communities in the human mouth are comprised of many different bacterial species. Most of them are commensal and essential to keep equilibrium in the mouth ecosystem. At the same time, some of them are directly involved in the development of oral diseases, such as cavities and periodontal disease [15, 21]. Thus, the accurate taxonomical composition of these species in health and disease will help us identify possible pathogenic species.

**Figure 8:**
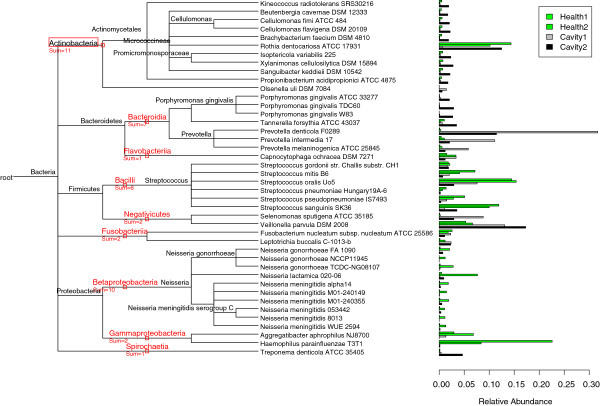
**Taxonomic composition of human oral microbiota.** The estimated relative abundance of genomes whose relative abundance is greater than 1% in at least one of four oral samples is shown in the bar plot, and the taxonomic tree structure of the detected genomes is attached accordingly. The four samples consists of two healthy control sets and two cavity sets. The cavity set labeled “Cavity1” is at an intermediate stage of cavity development, and the other “Cavity2” at an advanced stage.

We downloaded 4 sets of raw reads from the MG-RAST server: two healthy control sets and two cavity sets, which contain 454 pyrosequencing reads of 425 bp on average. The stages of cavity development for the two cavity sets are different: one at an intermediate stage and the other at an advanced stage [15]. The estimated relative abundance by TAEC for each genome whose relative abundance is greater than 1% is shown in Figure 8.

Bacilli, Betaproteobacteria, and Gammaproteobacteria are abundant in the healthy samples, while Actinobacteria, Bacteroidia, and Negativicutes are abundant in the diseased sample. This agrees with the previous findings [11, 15]. Generally, the detected members of the Actinobacteria class are abundant in the diseased group, especially for the second cavity sample which is at the advanced stage of disease. This finding is consistent with the conclusion of the paper [22]. An interesting observation is that *Rothia dentocariosa* is plenty in the healthy samples as well as in the advanced cavity sample. According to [23], *R. dentocariosa* is a largely benign gram positive microbe residing in human mouth but does very rarely cause disease, e.g., *Rothia* periodontal disease. Thus, it requires a further study with more samples to make a confirmative conclusion.

It also shows that all the detected members in the class Bacteroidia are abundant in the disease samples, including *Tannerella forsythia ATCC 43037* and 3 strains of *Porphyromonas gingivalis*, and 3 species of *prevotella*. This is consistent with the previous findings [24, 25], and it is well known that *Prevotella denticola* is a bacterial species found in the human mouth that causes infections of the oral cavity and adjacent structures [26]. We also noticed that the species *Leptotrichia buccalis* in the class of Fusobacteria is more abundant in the cavity samples than in the healthy controls, which is not surprised since it is the first species in the genus *Leptotrichia* found in human dental plaque [27]. The both detected species *Selenomonas sputigena* and *Veillonella parvula* in the class of Negativicutes are ample in the diseased samples. The fact that *Veillonella parvula* is gram-negative and normally occurs as a harmless parasite in the mouth cavities explains why we observe a large amount of *V. parvula* in both healthy and cavity samples [28]. Although it is considered non-pathogenic, *V. parvula* has been linked with rare cases of periodontal disease [28]. In addition, *S. sputigena* is the most frequently detected bacterial species in the genus of *Selenomonas* in the cavity/periodontal sample [29]. The species *Treponema denticola* which belong to the class of Spriochaetia has been identified from the oral cavity of humans [30].

In Figure 8 it shows that two strains, *Aggregatibacter aphrophilus NJ8700* and *Haemophilus parainfluenzae T3T1*, and the members of *Neisseria* are depleted in the cavity samples and the members of *Streptococcus* are less common in the cavity samples, which belong to Gammaproteobacteria, Betaproteobacteria and Bacilli, respectively. As for the strains in Betaproteobacteria their abundance could be due to biological variation or bias from sample collection since only one healthy control sample shows this pattern. For the *Streptococcus* strains in Bacilli and two species of *Aggregatibacter aphrophilus* and *Haemophilus parainfluenzae*, they are greatly bountiful in healthy oral samples. Actually they have been used as antagonistic microorganisms to control/reduce the adhesion of periodontal pathogens [31].

Of particular interest is the difference in relative abundance between the two cavity samples. The species of *prevotella* have very high relative abundance for the intermediate stage cavity sample, which is labeled as “Cavity1” in Figure 8, compared to the healthy control samples and even to the other cavity sample. Their active role in the early development of cavities is confirmed by the fact of they are oxygen tolerant [32]. Similarly, the abundance of *Prophyromansa gingivalis* and *Treponema denticola* in the advanced cavity sample can be explained by their anaerobic characteristic [32].

### Human gut datasets

**Figure 9:**
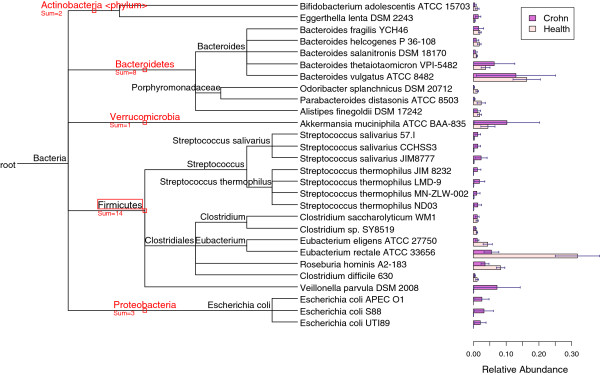
**Taxonomic composition of human gut microbiota.** The average relative abundance for the genomes (with greater than 1*%* in either health group or Crohn’s disease group) is plotted with the corresponding standard error in the bar plot, and the taxonomic tree structure of the detected genomes is attached accordingly.

The human gut is inhabited by a large number of bacterial species [33–35], and it is widely accepted that Crohn’s disease (CD) is associated with changes in microbial communities of human gut [16, 36]. We downloaded 11 sets of raw reads - seven healthy control sets and four CD sets - from the NCBI to estimate the difference in taxonomic composition between two groups [16]. The whole genome reads were produced by the Illumina, and the average length is 119 bp. The average estimated relative abundance by TAEC for genomes whose relative abundance is greater than 0.01 is shown in Figure 9.

It shows that the four major bacterial phyla in healthy people are Firmicutes, Bacteroidetes, Proteobacteria, and Actinobacteia, which agrees with the previous findings [11, 16, 36]. Interestingly, we detected another phylum - Verrucomicrobia - which is represented by a species *Akkermansia muciniphila* with relatively high abundance in both diseased and healthy samples. Actually, Verrucomicrobia can be occasionally observed in human gut [37].

The species *Eggerthella lenta* in the phylum Actinobacteia shows higher value in the CD patients than in the healthy controls, which is confirmed by the finding in a study of bacteremia for a CD patient [38]. Generally, the phylum Firmicutes depletes in the CD patients than in the healthy controls, largely due to the depletion of *Clostridiales* [39]. However, the genus *Steptococcus* shows a clear pattern that it is over represented in the CD patients, which concurs with the antigens findings in CD [40]. Meanwhile, the increase of *Veillonella parvula* in the CD patients can be confirmed by a metagenomic study of CD [41]. For the phylum Proteobacteria its increases in the CD patients is mainly due to the high relative abundance of three strains of *E. coli* [40, 41]. Regarding the phylum Bacteroidetes the findings on the CD patients are inconsistent [42]. Most studies showed that it is more prevalent in CD patients compared with healthy controls [42, 43]. By contrast, Frank et al. found that Bacteroidetes are significantly depleted in CD patients [44] by using q-PCR. Our analysis results are consistent with the later.

## Discussion

Many genomes share similarity in the genomic sequence, which is difficult to be captured from analyzing short sequence data alone. Currently, it is still very challenging to accurately estimate the taxonomic composition of a metagnomic sample containing similar species, without utilizing the genomic similarity. TAEC employs the similarity in the genomic sequence in addition to the quality of alignment to estimate the relative abundance, enabling accurate estimation of taxonomic composition at the species level in the taxonomy tree and even lower level if the depth of coverage is high enough.In addition to its accuracy, TAEC could provide a way to check the reliability of outputs: the estimated relative abundance along with the similarity between genomes allows us to identify which genomes are susceptible to high errors. For instance, if a genome shares very low similarity with other genomes in a sample, the accuracy of its estimated relative abundance is not affected by the relative abundance of the others. On the other hand, if a genome shares high similarity with other genomes, the accuracy of its estimated relative abundance is dependent on the relative abundance of the others and the depth of coverage as shown in Figure 7 and Additional file 4: Figure S2 and Additional file 5: Figure S3. Thus, with the information of relative abundance along with the similarity between genomes, we can narrow down which estimation of relative abundance is more reliable without any extra steps, like bootstrap suggested in TAMER.

TAEC has two limitations: 1) genomes that are not in a reference database cannot be correctly detected even if their true abundance is very high, and 2) very low abundant genomes, specifically their relative abundance is less than 0.05% of the genome with most abundance, cannot be detected regardless of their genomic similarities to the most abundant genome. The first limitation is common to all homology-based approaches, and generally the second limitation pose no problems since we usually are interested in genomes whose relative abundance is greater than 1%.

The length of reads in a real metagenomic sample varies, and this variation can change the number of reads assigned to a genome. However, the change mostly occurs on false genomes since the probability that a read originating from a genome can be assigned to the true genome is barely affected by the change in read length. Therefore, small variation in read length does not cause significant errors in the estimated relative abundance of possibly true genomes. However, a proper similarity matrix should be used for the accurate estimation. For instance, if the averaged length of reads is 110 bp, a similarity matrix created with the read length close to 110 bp should be used. It could cause significantly high errors, otherwise.

## Conclusion

TAEC is developed as a new homology-based approach to improve the estimation of taxonomic composition of metagenomic samples. Its performance is very consistent as demonstrated in various simulation studies. Particularly, it outperforms other existing methods when there exist closely related genomes in a sample. Moreover, it is also reliable in a sense that it could provide a way to check the reliability of outputs, which is critical in the analysis of many metagenomic projects, especially related to human health.

## Electronic supplementary material

Additional file 1: **Supplementary Notes: An example of the elimination algorithm.** An equivalent elimination algorithm in while loops. The selection of a similarity threshold and *K*_0_. (PDF 137 KB)

Additional file 2: **The choice of** ***α*** **value on various length of reads in terms of RRMSE.** (XLSX 61 KB)

Additional file 3: **Estimation accuracy comparison for three methods on the dataset with three** ***E. Coli*** **strains at the species level.** The performance of the three methods on the two samples that contain three *E. coli* strains, one at the same relative abundance ratio and the other at different relative abundance ratios, is compared by RRMSE as the number of false genomes in a reference database for GASiC increases. For TAMER and TAEC the reference database is kept same, i.e., NCBI bacteria database. (PDF 116 KB)

Additional file 4: **Estimation accuracy comparison for three methods on the dataset of three** ***E. Coli*** **strains, with relative abundance in the ratio of 1:10:20.** (PDF 97 KB)

Additional file 5: **Estimation accuracy comparison for three methods on the dataset of three** ***E. Coli*** **strains, with the same relative abundance ratio.** (PDF 96 KB)
