# Supplementary material for: Accurate genome relative abundance estimation for closely related species in a metagenomic sample
Source: BMC Bioinformatics. 2014 Jul 16;15(1):242. doi: 10.1186/1471-2105-15-242 (PMC4131027; doi:10.1186/1471-2105-15-242)
Supplement: Supplementary file 1 — Additional file 1: Supplementary Notes: An example of the elimination algorithm. An equivalent elimination algorithm in while loops. The selection of a similarity threshold and K 0. (PDF 137 KB) [file 12859_2014_6526_MOESM1_ESM.pdf]

Supplementary Notes

1. A simple example of the algorithm for the elimination stage

$$A_j = (A_{j-1} P_j S_j)_+ .$$

Let's consider the following alignment result:

Read ID	*Genome ID*	*Alignment Score*	*Read ID*	*Genome ID*	*Alignment Score*
R1	*G1*	*98*	*R4*	*G1*	*98*
R1	*G3*	*98*	*R4*	*G3*	*98*
R1	*G4*	*90*	*R5*	*G1*	*87*
R2	*G2*	*99*	*R5*	*G4*	*97*
R2	*G4*	*99*	*R6*	*G1*	*99*
R3	*G3*	*97*	*R6*	*G3*	*99*

In the elimination stage, each read is assigned to genome(s) with the highest alignment score. Thus,

$$A_0 = \begin{array}{c} \\ R1 \\ R2 \\ R3 \\ R4 \\ R5 \\ R6 \end{array} \begin{array}{c} \begin{array}{cccc} G1 & G2 & G3 & G4 \end{array} \\ \begin{pmatrix} 1 & 0 & 1 & 0 \\ 0 & 1 & 0 & 1 \\ 0 & 0 & 1 & 0 \\ 1 & 0 & 1 & 0 \\ 0 & 0 & 0 & 1 \\ 1 & 0 & 1 & 0 \end{pmatrix} \end{array} .$$

Since the third column a_3 denoted by $G3$ has the highest column sum, $a_{(1)} = \max_{1 \le \ell \le 4} ||a_\ell|| = a_3$. Thus, P_1 permutes the first column a_1 and the third column $a_{(1)} = a_3$, and then S_1 subtracts a_1 from a_2, a_3 and a_4. That is,

$$A_1 = (A_0 P_1 S_1)_+ = \left(\begin{array}{c} \begin{array}{cccc} G1 & G2 & G3 & G4 \end{array} \\ \begin{pmatrix} 1 & 0 & 1 & 0 \\ 0 & 1 & 0 & 1 \\ 0 & 0 & 1 & 0 \\ 1 & 0 & 1 & 0 \\ 0 & 0 & 0 & 1 \\ 1 & 0 & 1 & 0 \end{pmatrix} \end{array} \begin{pmatrix} 0 & 0 & 1 & 0 \\ 0 & 1 & 0 & 0 \\ 1 & 0 & 0 & 0 \\ 0 & 0 & 0 & 1 \end{pmatrix} \begin{pmatrix} 1 & -1 & -1 & -1 \\ 0 & 1 & 0 & 0 \\ 0 & 0 & 1 & 0 \\ 0 & 0 & 0 & 1 \end{pmatrix} \right)_+$$

$$= \left(\begin{array}{c} \begin{array}{cccc} G3 & G2 & G1 & G4 \end{array} \\ \begin{pmatrix} 1 & -1 & 0 & -1 \\ 0 & 1 & 0 & 1 \\ 1 & -1 & -1 & -1 \\ 1 & -1 & 0 & -1 \\ 0 & 0 & 0 & 1 \\ 1 & -1 & 0 & -1 \end{pmatrix} \end{array} \right)_+ = \begin{array}{c} \begin{array}{cccc} G3 & G2 & G1 & G4 \end{array} \\ \begin{pmatrix} 1 & 0 & 0 & 0 \\ 0 & 1 & 0 & 1 \\ 1 & 0 & 0 & 0 \\ 1 & 0 & 0 & 0 \\ 0 & 0 & 0 & 1 \\ 1 & 0 & 0 & 0 \end{pmatrix} \end{array} .$$

Now, $a_{(2)} = \max_{2 \le \ell \le 4} ||a_\ell|| = a_4$ so P_2 permutes the second column a_2 and the fourth column $a_{(2)} = a_4$, and then S_2 subtracts a_2 from a_3 and a_4. That is,

$$A_2 = (A_1 P_2 S_2)_+ = \left(\begin{pmatrix} 1 & 0 & 0 & 0 \\ 0 & 1 & 0 & 1 \\ 1 & 0 & 0 & 0 \\ 1 & 0 & 0 & 0 \\ 0 & 0 & 0 & 1 \\ 1 & 0 & 0 & 0 \end{pmatrix}^{\begin{matrix} G3 & G2 & G1 & G4 \end{matrix}} \begin{pmatrix} 1 & 0 & 0 & 0 \\ 0 & 0 & 0 & 1 \\ 0 & 0 & 1 & 0 \\ 0 & 1 & 0 & 0 \end{pmatrix} \begin{pmatrix} 1 & 0 & 0 & 0 \\ 0 & 1 & -1 & -1 \\ 0 & 0 & 1 & 0 \\ 0 & 0 & 0 & 1 \end{pmatrix} \right)_+$$

$$= \left(\begin{pmatrix} 1 & 0 & 0 & 0 \\ 0 & 1 & -1 & 0 \\ 1 & 0 & 0 & 0 \\ 1 & 0 & 0 & 0 \\ 0 & 1 & -1 & -1 \\ 1 & 0 & 0 & 0 \end{pmatrix}^{\begin{matrix} G3 & G4 & G1 & G2 \end{matrix}} \right)_+ = \begin{pmatrix} 1 & 0 & 0 & 0 \\ 0 & 1 & 0 & 0 \\ 1 & 0 & 0 & 0 \\ 1 & 0 & 0 & 0 \\ 0 & 1 & 0 & 0 \\ 1 & 0 & 0 & 0 \end{pmatrix}^{\begin{matrix} G3 & G4 & G1 & G2 \end{matrix}}.$$

Since $||a_{(3)}||_1 = 0$, we stop and remove the columns a_3 and a_4, which correspond to $G1$ and $G2$. That is, we assume the presence of these genomes is due to their similarity to $G3$ and $G4$ since there is no reads uniquely assigned to them.

2. An equivalent algorithm for the elimination stage using while loop

$j = 1$
find $a_{(j)}$
colsum = sum of the elements of $a_{(j)}$
while (colsum > 0)
    store the genome ID corresponding to $a_{(j)}$
    permute a_j and $a_{(j)}$
    subtract a_j from a_k, where $k = j+1, j+2, \ldots, N$
    if $a_{ik} < 0$, $a_{ik} = 0$, where $i = 1, 2, \ldots, K$ and $k = j+1, j+2, \ldots, N$
    $j = j + 1$
    find $a_{(j)}$
    colsum = sum of the elements of $a_{(j)}$

3. The selection of a threshold for the similarity among genomes and the number of reads K_0 used to estimate the similarity

Our selection of the similarity threshold was closely related to our selection of K_0: we first simulated 5000 reads from a genome and computed the similarity between the genome and other genomes. We repeated 10 times with the same genome. In the 10 repetitions we observed a few genomes to have the similarity less than 0.005 in some simulations but 0 in the others. We also repeated with four other genomes and observed the similar results. We repeated the whole procedures with K_0 = 10000, 30000, 50000 and observed a few genomes still having low similarity in some simulations but 0 in the others. Their similarity, however, was less than 0.001 with these values of K_0. Since we could not tell whether the similarity between two genomes was due to the true similarity or systematic error if their similarity is less than 0.001, we treated this low similarity as a systematic error and set 0.001 as the similarity threshold. In general, we obtain a more accurate estimation with a higher sample size, but the gain becomes almost negligible when sample sizes are high enough. We conservatively selected $K_0 = 30000$ instead of $K_0 = 10000$.
