# Supplementary figures and images for: Accurate genome relative abundance estimation for closely related species in a metagenomic sample
Source: BMC Bioinformatics. 2014 Jul 16;15(1):242. doi: 10.1186/1471-2105-15-242 (PMC4131027; doi:10.1186/1471-2105-15-242)

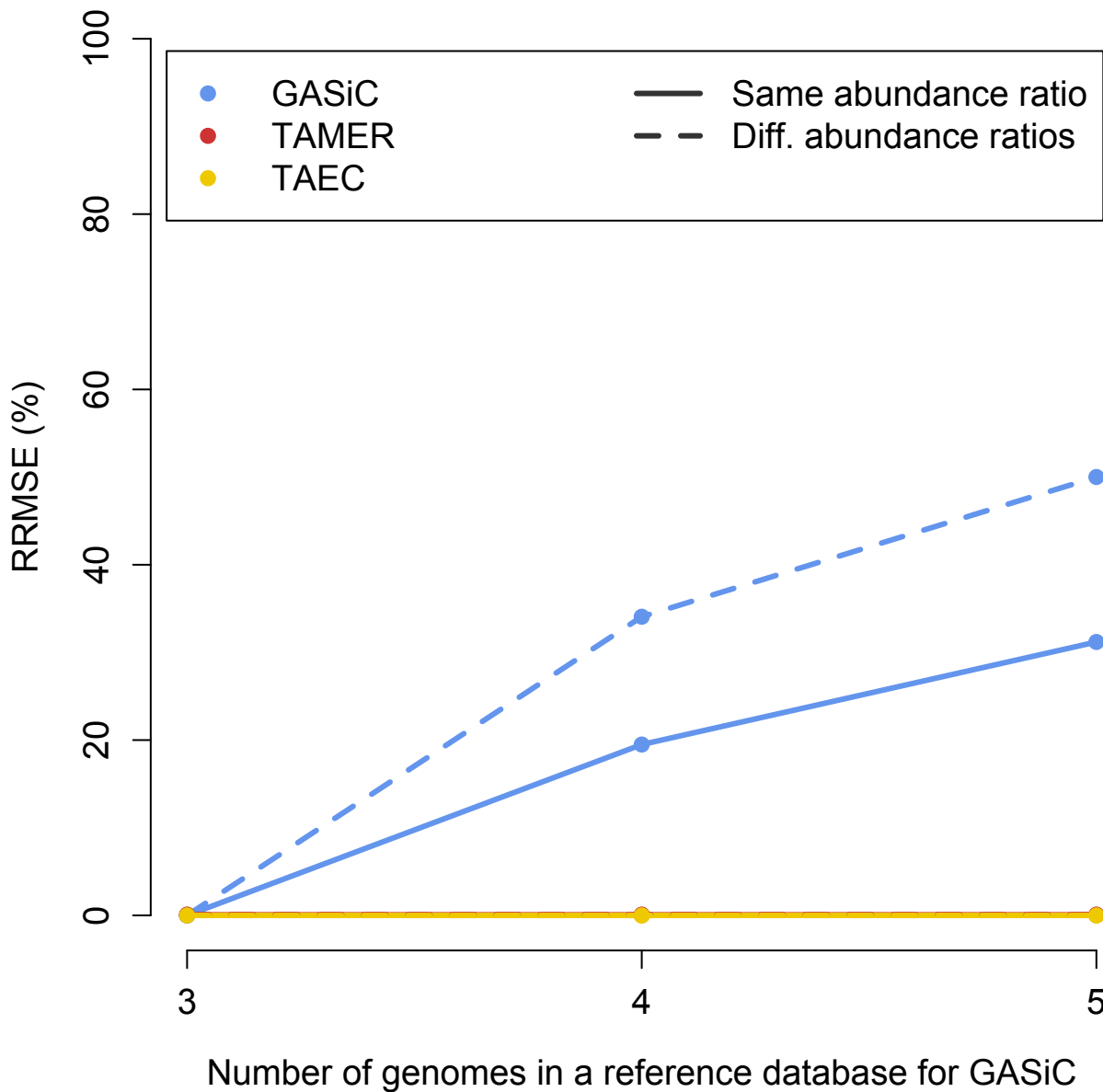

Supplement: Supplementary file 3 — Additional file 3: Estimation accuracy comparison for three methods on the dataset with three E. Coli strains at the species level. The performance of the three methods on the two samples that contain three E. coli strains, one at the same relative abundance ratio and the other at different relative abundance ratios, is compared by RRMSE as the number of false genomes in a reference database for GASiC increases. For TAMER and TAEC the reference database is kept same, i.e., NCBI bacteria database. (PDF 116 KB) [file 12859_2014_6526_MOESM3_ESM.pdf]

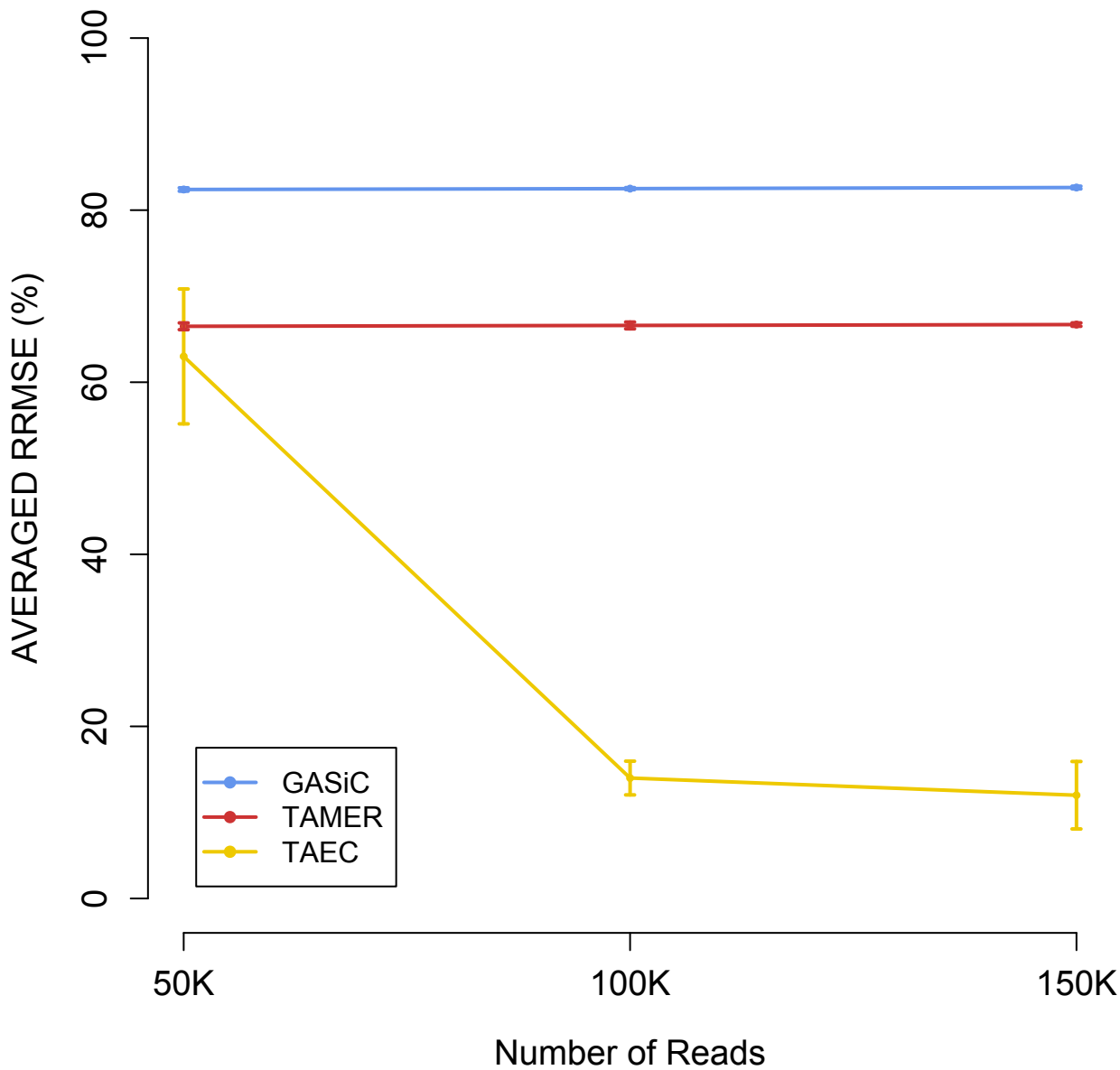

Supplement: Supplementary file 4 — Additional file 4: Estimation accuracy comparison for three methods on the dataset of three E. Coli strains, with relative abundance in the ratio of 1:10:20. (PDF 97 KB) [file 12859_2014_6526_MOESM4_ESM.pdf]

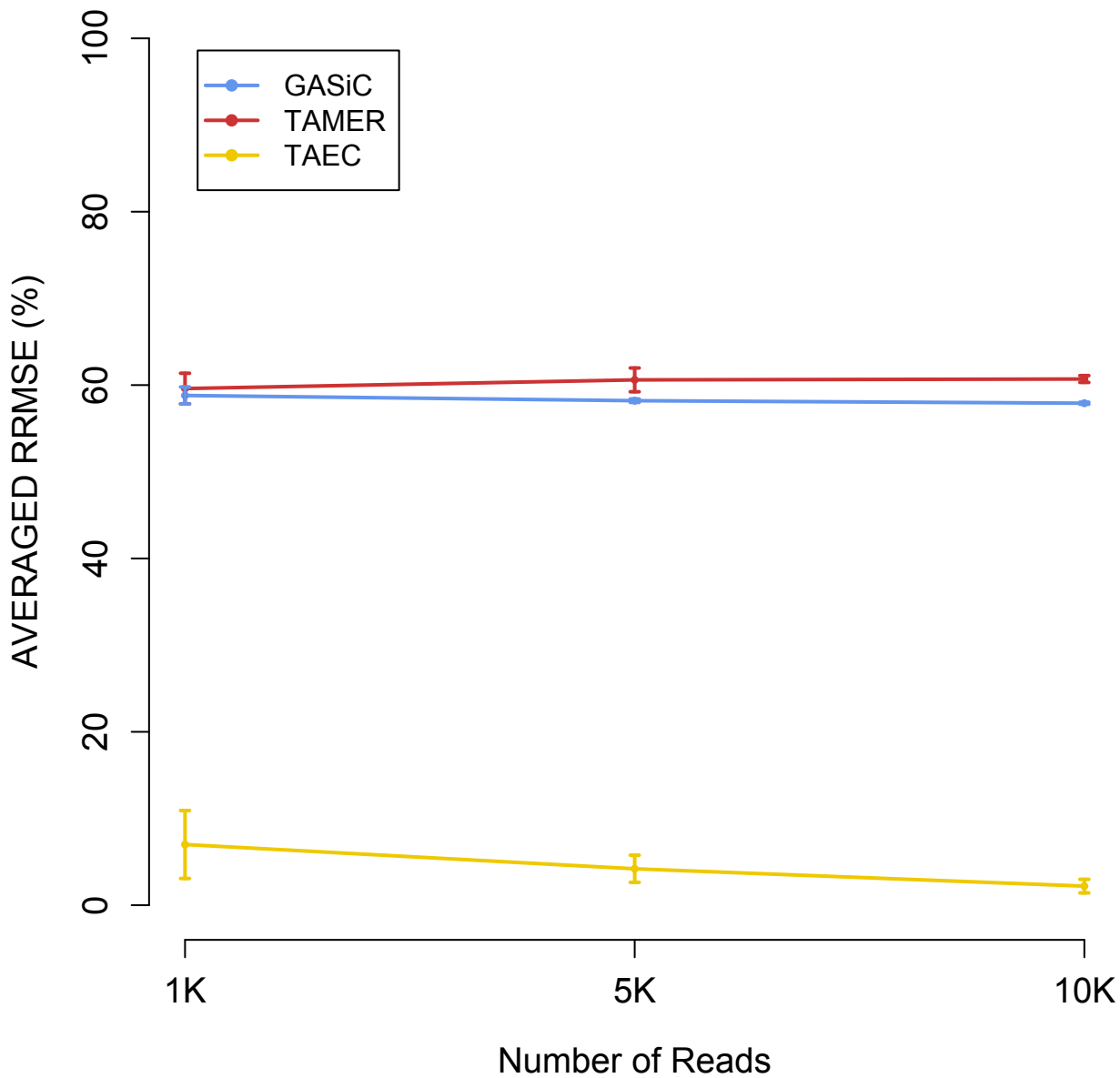

Supplement: Supplementary file 5 — Additional file 5: Estimation accuracy comparison for three methods on the dataset of three E. Coli strains, with the same relative abundance ratio. (PDF 96 KB) [file 12859_2014_6526_MOESM5_ESM.pdf]
